# Personality Determinants of Success in Men’s Sports in the Light of the Big Five

**DOI:** 10.3390/ijerph18126297

**Published:** 2021-06-10

**Authors:** Paweł Piepiora, Zbigniew Piepiora

**Affiliations:** 1 Faculty of Physical Education and Sport, University School of Physical Education in Wrocław, 51-612 Wrocław, Poland; 2 Faculty of Environmental Engineering and Geodesy, Wrocław University of Environmental and Life Sciences, 50-375 Wrocław, Poland; zbigniew.piepiora@upwr.edu.pl

**Keywords:** sport psychology, personality, neuroticism, champions

## Abstract

The aim of the study is to describe personality profiles and determinants of success in sports in relation to the Big Five Personality Model. In order to achieve this aim, personality profiles of players from various sports disciplines was set against the personality profile of champions—players who are considerably successful in sports competitions. Subsequently, an attempt was made to determine which personality traits significantly determine belonging to the group of champions—and therefore determine success in sport. The participants were men aged between 20 and 29 from the Polish population of sportsmen. A total of 1260 athletes were tested, out of whom 118 were qualified to the champions sample—those athletes had significant sports achievements. The research used the NEO-FFI Personality Questionnaire. Basic descriptive statistics, a series of Student’s *t*-tests for independent samples using the bootstrapping method, as well as a logistic regression model were performed. In relation to other athletes, champions were characterized by a lower level of neuroticism and a higher level of extraversion, openness to experience, agreeableness, and conscientiousness. An important personality determinant was neuroticism: the lower the level of neuroticism, the greater the probability of an athlete being classified as a champion. There are differences between champions and other athletes in all personality dimensions in terms of the Big Five. Based on the result of the research, it can be stated that personality differences should be seen as a consequence of athletes’ success, rather than as a reason for athletes’ success, based on their age between 20 and 29.

## 1. Introduction

A problem that has long been of interest to sports psychologists, coaches, and athletes alike concerns the determination of the personality traits of a champion [1,2]. This particular task would involve the identification of the athletes’ personality traits which are essential to their success in sport [3,4].

For instance, Garland and Barry [5] carried out an experiment on American college athletes, varying in terms of physical fitness and sport level, to test the relationship between personality as measured by the 16-Factor Personality Questionnaire and their sports performance. It was shown that personality traits such as belief rigidity, extraversion, group dependence, and emotional stability were responsible in 29% for variations in physical fitness. Davis [6], in turn, tried to predict the success of professional hockey players by measuring their personality traits, but found no correlation. He believed that success was influenced by more important psychophysical factors.

In another study, Lerner and Locke [7] measured the willingness of American college athletes to compete in relation to their achievement motivation. To this end, they used the Sports Orientation Questionnaire, and measured their endurance by performing squats. Similarly, as in Garland and Barry [5], a relationship was found between personality and success. Psychological factors such as goal setting and self-efficacy have been shown to validate the influence of personality on athletic performance.

In a cutting-edge experiment by Piedmont, Hill, and Blanco [8], four different Division 1 NCAA soccer teams were tested with the Big Five model. Coach ratings for several dimensions of player performance and actual game statistics were also collected. Regression analysis indicated that personality dimensions of neuroticism and conscientiousness explained about 23% of the coaches’ variance ratings, while conscientiousness was the only predictor of actual game statistics, explaining about 8% of the variance.

A slightly different research was carried out by McKelvie, Lemieux, and Stout [9] on groups of university athletes (divided into contact and non-contact disciplines) and non-athletes, with the use of the Eysenck Personality Inventory. Extraversion did not differ significantly between athletes and non-athletes, nor between contact and non-contact sportsmen, but was higher for athletes in general compared to American academic standards. In the case of neuroticism, successful athletes scored significantly lower than unsuccessful athletes. As neither extraversion nor neuroticism results has changed over the four years of continuous research, one might conclude that people with higher extraversion and lower neuroticism are interested in academic sports.

In another study Anghel, Banica, and Ionescu [10] found out that personality traits of elite athletes were dependent and distinctive of the sports discipline they trained. The athletes were characterized by low neuroticism, high extraversion, and conscientiousness, but the intensity of individual personality traits depended on the trained sport discipline. This indicates the existence of a general personality profile of athletes, in which the strength of the acceleration of personality traits is determined by particular sports disciplines.

Mirzaei, Nikbakhsh, and Sharififar [11] made further attempts to investigate the relationship between personality traits and sports performance in the Big Five model. The research sample included more than 200 non-elite soccer players and futsal soccer players. It was shown that among the personality traits, only conscientiousness had a significant correlation with sports performance—conscientiousness alone was the only predictor of sports performance.

Then, Kim, Gardant, Bosselut, and Eys [12] conducted an experiment on a sample of team sports players and showed that low neuroticism, high extraversion, and conscientiousness all influence informal role-taking in a sports team, depending on the sports team. The same year, Steca, Baretta, Greco, D’Addario, and Monzani [13] examined more than 800 athletes and non-athletes with the use of the Big Five model. It was shown that the most successful athletes in their discipline had higher scores than the non-athletes in every dimension of the Big Five, except neuroticism, in which they scored lower. In contrast, less successful athletes outperformed the non-athletes only in extraversion and agreeableness. Athletes who were more successful in their competitive sports (champions) showed greater emotional stability (lower neuroticism), extraversion, openness to experience, agreeableness, and conscientiousness than less effective athletes. Moreover, individual athletes turned out to be more energetic and open-minded than team athletes. In another study, Piepiora and Witkowski [14] tried to generate psychological personality profiles of athletes performing individual and team disciplines, depending on the type of pressure exerted on the opponent in the starting situation. Differences were found in the scales of neuroticism and conscientiousness between sports disciplines in which pressure is exerted indirectly on the opponent, and disciplines in which the pressure exerted directly on the opponent. The study groups, with the exception of volleyball players and football players, differed from each other in terms of neuroticism scale, while the volleyball players showed less agreeableness and conscientiousness than other athletes.

Taking the above research and reflections as the starting point for the research problem formulation, it should be assumed that personality conditioning in sports champions in relation to the population of unsuccessful athletes, according to the Big Five model, focuses on lower neuroticism and higher extraversion, openness to experience, agreeableness, and conscientiousness [15,16]. However, there is ambiguity in relation to the type of sport, competing classes, or cultural affiliations. Personality traits are adequate to the specificity of the trained sports discipline, and its goals and challenges. The personality profiles of the athletes are at similar levels, but they are not identical. Among athletes, it is extremely difficult to distinguish and define the most favorable type of personality, as it is largely influenced by the trained sports discipline, and it determines the personal conditions of athletes [17,18,19,20]. Therefore, it was deemed necessary to verify which personality traits, and to what extent said traits, define sports champions and determine success in sports.

The research problem was an attempt at defining personality profile of sports champions and personality determinants of success in sport in the light of the Big Five factor model. In connection with the above, personality profiles of players from various sports disciplines in the areas of combat sports [21], individual sports [22], and team sports [23] were compared with the personality profile of champions [16]—players who are very successful in sports rivalry. Subsequently, attempts were made to determine which personality traits significantly determine belonging to the group of champions—and thus determine success in sport. For this purpose, the Big Five model was used, as it does not transgress the definition of personality traits understood as behavioral properties, showing interindividual variability and intra-individual temporal and situational permanence. They adopt a number of methodological assumptions that define the status of personality traits as “basic” dimensions of personality. The Big Five model defines the most general characteristics of behavior that are actual, invariant, universal, and biologically conditioned [24].

## 2. Methodology

### 2.1. Participants

The research was carried out between 1 October 2015 and 30 September 2019. The subjects of the study were men, intentionally, non-randomly selected from the Polish population of sportsmen. The criteria for the non-random, purposeful selection of respondents were: free will to participate in the study; senior age (between 20 and 29 years of age); at least the second or higher sports class; many years of sports experience—three years or more; a current competition license; and documented sports achievements at various levels of rivalry (national, continental, and world). A total of 1260 competitors were tested, 30 each from the following sports disciplines: alpine skiing, American football, archery, athletics—long runs, athletics—short runs, ballroom dancing, basketball, beach volleyball, biathlon, bodybuilding, Brazilian jiu-jitsu, break dance, canoeing, cycling, fitness, floorball, football, futsal, handball, horse riding, indoor volleyball, judo, ju-jitsu, kickboxing, kyokushin karate, mixed martial arts, mountaineering, Olympic karate, orienteering, Oyama karate, rugby, shidokan karate, shotokan karate, snowboarding, sport climbing, sport shooting, swimming, taekwondo, tennis, tobogganing, ultimate frisbee, and wrestling. Such a distribution of disciplines depended on the respondents’ willingness to participate in the study. From the above population, 118 athletes were qualified to the sample of champions. Players with international sports successes were defined as champions. Therefore, the criterion for qualifying Polish players to the sample of champions was their 1st, 2nd, or 3rd place in international sports competitions. This includes medalists of the World Championship, the European Championship, the World Cup, the European Cup, the World Games 2017, and other ranked international tournaments in their sports disciplines. The following champions with significant sports achievements were identified: from alpine skiing (3), archery (5), ballroom dance (2), beach volleyball (2), biathlon (4), bodybuilding (4), Brazilian jiu jitsu (4), break dance (2), canoeing (2), cycling (2), equestrian (1), fitness (4), floorball (2), futsal (2), ju jitsu (5), judo (3), kickboxing (4), kyokushin karate (6), mixed martial arts (4), mountaineering (1), Olympic karate (1), orienteering (3), Oyama karate (4), shidokan karate (5), short (2) and long runners (8), shotokan karate (6), snowboard (3), sports climbing (3), swimming (3), taekwondo (5), target shooting (1), toboggan (3), volleyball (7), and wrestling (2). The other 1142 athletes were sportsmen with only national (Polish) sports successes. Only the best results of the respondents on the day of the study were included in the study. The achievements of already tested players have not been updated.

### 2.2. Method

The NEO-FFI Personality Questionnaire was selected to examine the athletes’ personality in terms of the Big Five factor model [25]. The selection criterion was justified by: the location of NEO-FFI in the theoretical model and relatively large methodological formalization compared to other approaches developed within the five-factor personality model; good psychometric characteristics; rich factual documentation of the measurement accuracy for the factors of the original version, which allows to assume that the inventory may be useful in scientific and practical research; and duration time acceptable for the athletes.

The items of the NEO-FFI Personality Questionnaire are formed by five scales measuring the factors of the Big Five model. They are marked with abbreviations of the first letters of the factors: neuroticism, extraversion, openness to experience, agreeableness, and conscientiousness. For the purposes of this study, the acronym NEOAC was adopted, i.e., the above-mentioned sequence of factors.

The NEO-FFI Personality Questionnaire is internally consistent. Its validity was demonstrated on the basis of research on the relationship between the results of the questionnaire and the assessments of the subjects made by observers, the heritability of the measured traits, and their correlation with other dimensions of personality and temperament. The factor validity was also verified. The results allow for a full description of the respondents’ personality in the five-factor approach of the Big Five and forecasting their adaptation possibilities to the professional environment [24,25]. Moreover, the NEO-FFI assumes a maximum examination time of one hour. Such duration of the study was acceptable to athletes who expressed free will to participate.

### 2.3. Data Analysis

In order to verify the research problem, statistical analyses were performed using the IBM SPSS Statistics, version 25 (IBM Polska, Warsaw, Poland). Beforehand, basic descriptive statistics were calculated for each sports discipline included in the study. It was decided not to calculate normal distribution tests for each personality trait in each discipline due to the relatively small sample size and the multiple comparisons. Both of these factors could render the conclusions drawn from the results of such tests incorrect. For this reason, the so-called the rule of thumb was used for the analysis of skewness value. If the skewness value for a given variable ranged from −2 to 2, then it could be concluded that the distributions of these variables are not too asymmetric, which allows for the use of parametric tests. In the case of differently classified data comparisons, the skewness values for the compared groups were checked before the analysis. Each time, they fell within the accepted range. In order to solve the research problem, Student’s *t*-tests for independent samples and a logistic regression model were performed. This model presents an exploratory analysis to see how individual personality traits will predict belonging to the champion group. It was necessary as *t*-tests only verify differences in a single dimension.

### 2.4. Procedure

All respondents consented to the processing of data related to their participation in the research by the researcher.

The project received a positive opinion (number 20/2019) of the Senate Committee on Ethics of Scientific Research at the University School of Physical Education in Wrocław.

## 3. Results

The sample of champions consisted of 118 men (9% of the respondents), and the sample of other athletes, 1142 men (91% of the respondents). In order to verify the research problem, a number of Student’s *t*-tests were carried out for independent samples using the bootstrapping method, set at 10,000 samples and a 95% confidence interval. Five Student’s *t*-tests were performed, and the statistical significance level for the analyses of variance was calculated as α = 0.01.

**Figure 1:**
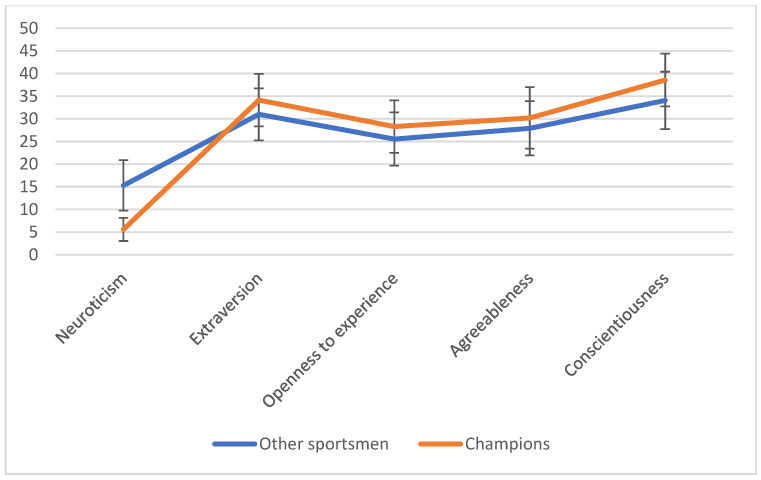
Line graph of personality profiles; breakdown into champions and other athletes.

The test results showed statistically significant differences in all personality traits from the Big Five model. In the case of neuroticism, a very strong difference effect persisted. A moderately strong effect was observed for extraversion and conscientiousness, and weak effects were observed for openness to experience and agreeableness. Sports champions were characterized by a lower level of neuroticism and a higher level of extraversion, openness to experience, agreeableness, and conscientiousness than the group of other athletes. The exact values of the performed tests are presented in Table 1. The samples are presented graphically in Figure 1.

Finally, in order to verify the analyzed results, a logistic regression model was prepared where, based on personality traits, an attempt was made to classify the respondents into the group of sports champions and other athletes.

In the first step, all personality traits were introduced as predictors of the athletes’ level. The null model was characterized by 90.6% correct classifications, which results from the ratio of the number of other athletes to all research subjects. The classification threshold, based on the ROC analysis, was set to 0.7. The model with five predictors was statistically significant χ^2^ (5) = 425.68; *p* < 0.001, and Nagelkerke’s pseudo-R^2^ was 0.62, which means that the proposed model explains about 62% of the variance. The Hosmer–Lemeshow goodness of fit test was statistically insignificant χ^2^ (8) = 7.49; *p* = 0.485. The entire model correctly classified 94.3% of the observations. The analysis of the significance of the predictors in the discussed model showed that only neuroticism significantly predicted belonging to the champions group or to the other athletes group. For this reason, another model was created in which neuroticism was the only predictor. The second model was statistically significant χ^2^ (1) = 423.02; *p* < 0.001, and Nagelkerke’s pseudo-R^2^ was 0.62. The goodness of fit test was again statistically insignificant χ^2^ (7) = 13.44; *p* = 0.062. The overall percentage of correct classifications was also 94.3%. Pseudo-R^2^ for one personality variable of logistic regression was 62% of the variance as other athletes are very different from the champions in their level of neuroticism. In the *t*-test analysis, the effect size was *d* = 1.81, which is a very high result. It is rarely seen, but apparently the two groups are quite different in this respect. The other personality measures did not contribute to the percentage of explained variance. Therefore, the second model, with the only predictor being the neuroticism measure, turned out to be as good as the model with five predictors. This means that neuroticism was the key personality trait that predicted the level of achievement among the tested athletes. A relationship was established in the developed model: the lower the level of neuroticism, the greater the probability of being classified as a sports champion. The relationship is presented in Table 2.

## 4. Discussion

The analyses showed statistically significant differences in all personality dimensions in the Big Five five-factor approach; namely: sports champions were characterized by a lower level of neuroticism and a higher level of extraversion, openness to experience, agreeableness, and conscientiousness than other athletes. This personality profile of sports champions confirmed earlier research reports [16,21,22,23,26], and at the same time negated the research of Mirzaei and colleagues [11], which suggested that only high conscientiousness correlated with sports results.

Whether the personality determinants of success in sport were formed solely in the course of many years of sports career, or already at the beginning of sports practice still remains an open question. Therefore, the opinion of respected scientists such as Allen [17,18,19,20], or Vealey [27] cannot be ruled out. Factors disrupting or supporting the development of a young athlete are created by his immediate environment. This, in turn, is expressed in self-esteem, which has a significant impact on the shaping of the personality and competences of talented players.

The logistic regression model analyzed the obtained results. On the basis of the five-factor personality model, attempts were made to classify the researched population into the group of sports champions or the other athletes group. The research results have shown that neuroticism was an important personality trait, allowing to classify athletes according to their level of sports achievements; the lower the level of neuroticism, the greater the probability of being classified as a sports champion. The numerous relationships found in the research between personality dimensions and athletes in various randomizations allow us to conclude that the results concerning neuroticism as a determinant of personality success in sport are highly probable and may be universal. The only predictor of sports results, and thus a personality determinant of success in sports, in terms of the Big Five, was neuroticism.

The dimension of neuroticism reflects emotionality in terms of experiencing negative emotions, i.e., emotional adaptation in relation to emotional imbalance. The sports champions were distinguished by very low neuroticism, thus it can be assumed that they were emotionally stable, calm, relaxed, and able to deal with stress without experiencing anxiety, tension, and irritation; whereas other athletes had a higher level of neuroticism compared to the champions. This means that their negative emotions influenced their adaptation to the environment. Neurotic people were prone to irrational ideas, and relatively inadequate to control their drives and cope with stress. This is due to the general excitability of the vegetative system. The reactions are too great in relation to the strength of the acting stimuli. Emotionally unstable competitors experience very strong pre-start conditions and can collapse in the face of important competitions. It can be expected that in difficult situations, their efficiency of perception, speed and accuracy of sensorimotor responses, efficiency of thinking processes, and the quality and effectiveness of action will deteriorate significantly. The dimension of neuroticism includes six formally distinguished components: anxiety, aggressive hostility, depression, impulsiveness, hypersensitivity, and excessive self-criticism. Therefore, champions may be distinguished from other sportsmen by low level of anxiety, which has a positive effect on motivation [6]; low aggressive hostility that triggers the state of start readiness, which translates into the control of arousal before and during the competition, and bravery understood as fighting until the very end [28]; low depressiveness that indicates an optimistic mood and a positive attitude [29]; low impulsiveness that crystallizes emotion control [30]; low hypersensitivity that gives good concentration of attention and the need for strong sensory impressions, as well as the ability to cope with failure and experience success [31]; and finally, low self-criticism that determines self-confidence and self-efficacy [32].

Taking the above into consideration, the greatest cognitive value of this paper is to prove that neuroticism is an important personality condition for success in sport. Therefore, one should adopt broad perspectives of analyses of neuroticism components as mental determinants of sports success. As there is no data regarding whether social factors influence the personality of the surveyed sportsmen, one should also pay attention to the role of the social environment of sportsmen. This knowledge may be useful in the detection and proper development of sports talents, modernization of sports training and better adaptation of athletes to the environment after the end of their career. It is also important to notice that sports activities shape the personality of players [1,2,3,4,33]. Therefore, the differences in personality shown in this study can be seen as a consequence of the athletes’ success, rather than as a reason for athletes’ success, based on their age between 20 and 29. Sports activity could be seen as a self-confidence generator. Under the influence of trainings, adepts start to improve in a given discipline, and this moderates their personalities. Athletes become convinced that they are the authors of their own fate and that they create their own lives. This is why the successes achieved by the players build strong personalities of athletes.

The obtained research results also provide a new argument about the health aspects of sports training (in the context of health through rational, long-term sports training) in personality development. There are few empirical studies on the relationship of motor, technical and tactical training, and the results of personality tests. Hence, the possibilities of a broader interpretation of research results from an interdisciplinary perspective are limited.

At this point, the strengths and limitations of the conducted cognitive experiment should be equally noted. The research sample was homogeneous in terms of ethnicity, gender, and the age range of 20–29 years. Athletes of other nationalities, women, and other age groups were not included. The research was conducted on a large group of respondents from sports disciplines popular in Poland. However, it was not possible to examine athletes from all sports disciplines trained in Poland. The group of champions included Polish athletes with international sports successes. Therefore, the obtained research results can only be applied to a specific population of athletes. Thus, the following conclusion can be drawn: a low level of neuroticism is a personality determinant of success in sport among Polish male athletes between the ages of 20 and 29. However, one must bear in mind that the personality determinants of success in sport in various disciplines are distinct. This is due to the specificity of sports competition in martial arts [21], individual [22], and team [23] sports, as well as different psychological requirements they place on competitors [1,33].

However, the general personality profile of athletes in terms of the Big Five is low neuroticism, high extraversion and conscientiousness, average openness to experience and agreeableness [4,17]. In comparison with the reports by Allen [20], it was noticed that low neuroticism also has a significant role in the personality differentiation of champions from the rest of the athletes. It has been proven that a low level of neuroticism may be a personality determinant of sport success among Polish athletes between the ages of 20 and 29, and its intensity depends on the sports discipline. It is therefore suggested that the coaches analyze the personality conditions of the players for sports competition, as these have a significant impact on the sports results. Hence, in sports theory, one should adopt broad perspectives of personality component analyzes as mental determinants of sports success.

## 5. Conclusions

There are differences between champions and other athletes in all personality dimensions in terms of the Big Five. Sports champions were characterized by a lower level of neuroticism and a higher level of extraversion, openness to experience, agreeableness, and conscientiousness in relation to other athletes. Analysis of the obtained data by the logistic regression model proved that only neuroticism was an important personality determinant predicting the level of achievement among the studied athletes: the lower the level of neuroticism, the greater the probability of classifying the athlete to the champion group. Champions are presumably balanced and usually resistant to stress. They are not very sensitive to various stressors. They have better attention span, and they do not panic in difficult situations. Their well-being is stable, and their emotional reactions are adequate to the stimuli. Therefore, sports development of athletes without the knowledge of the specific features and personality structure of various sports representatives may be an artificial and ineffective activity. It remains an open question whether the personalities of the champions were shaped only in the course of many years of their sports career, or whether they already distinguished champions at the beginning of their sports practice. Therefore, based on the result of the research, it can be argued that personality differences should be seen as a consequence of the athletes’ success, rather than as a reason for the athletes’ success, based on their age between 20 and 29.

## Figures and Tables

**Table 1:** Analysis of differences between champions and other athletes in the intensity of personality traits.

	Other Sportsmen (*n* = 1142)		Champions (*n* = 118)				
Variables	M	SD	M	SD	*t*	*p*	Cohen’s *d*
Neuroticism *	15.30	5.58	5.58	2.56	33.79	<0.001	1.81
Extraversion	30.98	5.75	34.13	5.80	−5.65	<0.001	0.55
Openness to experience	25.54	5.87	28.28	5.79	−4.83	<0.001	0.47
Agreeableness	27.91	5.99	30.20	6.78	−3.91	0.001	0.38
Conscientiousness	34.06	6.33	38.56	5.82	−7.40	<0.001	0.72

* correction for heterogeneity of variance; *t*—t statistic value; *p*—significance level; and Cohen’s *d*—a measure of the size of the effect.

**Table 2:** Coefficients of the logistic regression model predicting classification to sports champions group or other athletes group, based on personality traits.

Explained Variable		*β*	*β SE*	*Wald’s χ* ^2^	*p*	*e^β^*	*R* ^2^
Other sportsmen vs. Champions	(Constant)	2.21	1.38	2.57	0.109	---	0.62
	Neuroticism	−0.67	0.06	124.99	<0.001	0.51	
	Extraversion	0.02	0.3	0.79	0.375	1.02	
	Openness to experience	−0.01	0.02	0.06	0.807	0.99	
	Agreeableness	<0.01	0.02	<0.01	0.952	1.00	
	Conscientiousness	0.03	0.02	1.23	0.267	1.03	
Other sportsmen vs. Champions	(Constant)	3.96	0.44	81.34	<0.001	---	0.62
	Neuroticism	−0.68	0.06	136.64	<0.001	0.51	

β—non-standardized Beta coefficient; β SE—standard error for the Beta coefficient; Wald’s χ^2^—chi-square statistics for Wald test; e^β^—odds ratio; and R^2^—statistics of model fit to data (R^2^ × 100%—percentage of explained variance).

## Data Availability

The authors confirm that the data supporting the findings of this study are available within the article.
